# Status of HIV vaccine development: progress and promise

**DOI:** 10.1002/jia2.26489

**Published:** 2025-05-15

**Authors:** Barton F. Haynes, Kevin O. Saunders, Beatrice H. Hahn, Kevin Wiehe, Lindsey R. Baden, George M. Shaw

**Affiliations:** ^1^ Duke Human Vaccine Institute Department of Medicine Duke University Durham North Carolina USA; ^2^ Duke Human Vaccine Institute Department of Surgery Duke University Durham North Carolina USA; ^3^ Departments of Medicine and Microbiology University of Pennsylvania Philadelphia Pennsylvania USA; ^4^ Department of Medicine Brigham and Women's Hospital, Harvard Medical School Boston Massachusetts USA

1

HIV Vaccine Awareness Day (HVAD) each year commemorates President Bill Clinton's 1997 declaration that “only a truly effective preventive HIV vaccine can limit and eventually eliminate the threat of AIDS.” Here, we review recent progress that the HIV vaccine field has made in inducing protective broadly neutralizing antibodies (bnAbs) that can prevent HIV acquisition. Several papers provide further review and discussion of the concepts discussed in this Viewpoint [1, 2, 3].

An HIV bnAb‐based vaccine has been difficult to develop because of the extensive genetic variability of HIV, its heavily glycosylated and conformationally masked envelope (Env) surface protein and the need to induce durable high levels of multiple bnAb specificities to achieve protection [4]. In addition, because HIV mutates so rapidly, it will be necessary to induce multiple types of bnAbs to fully cover the broad range of variants.

One solution to inducing naturally disfavoured bnAbs is to design immunogens that target naïve bnAb B cell precursors, expand them and select for improbable mutations that are roadblocks for bnAb affinity maturation [5, 6]. Following naïve B cell priming, sequential immunization with Env immunogens with increasing affinities will be needed to mature bnAb lineages along desired pathways [5, 7]. Thus, iterative vaccine design in animal models and in small Phase I clinical trials is required to assess the many steps in such a complex vaccine strategy. Such trials in the HIV Vaccine Trials Network (HVTN) are called Discovery Medicine trials [8]. Figure 1 shows the bnAb target epitopes on the HIV envelope for which a degree of success in inducing B cell lineages has been achieved by vaccination in immunoglobulin humanized mice, non‐human primates or humans. What follows here are brief updates on trials that have initiated immunization with bnAb B cell lineages primarily in either non‐human primates or in humans by vaccination.

**Figure 1:**
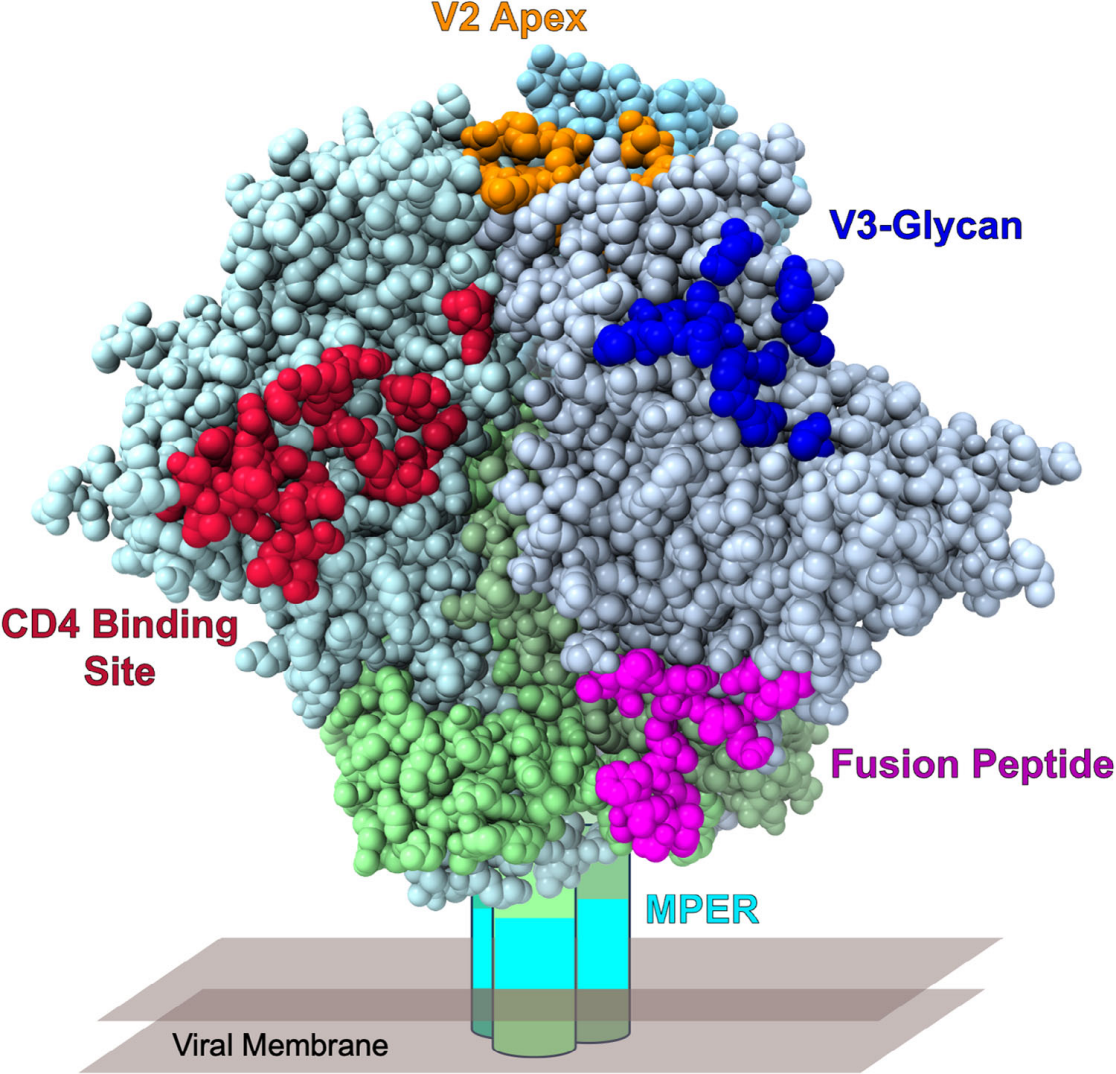
Classes of BNAb epitopes on HIV‐1 envelope. An effective HIV‐1 effective will require elicitation of a polyclonal broadly neutralizing antibody response to multiple sites of vulnerability on the Env trimer.  Conserved epitopic sites recognized by classes of known HIV‐1 bnAbs are shown in colour on the Env trimer (gp120 is coloured in shades of light blue; gp41 is coloured in shades of light green).

Gp41 membrane proximal external region (MPER) bnAbs are among the most broadly reactive HIV antibodies. An MPER peptide‐liposome priming immunogen designed to mimic gp41 bnAb binding sites and bind to a prototype bnAb naïve B cell receptor was used in the HVTN 133 clinical trial. B cells were induced that bound to the proximal MPER bnAb epitope—the most potent of these antibodies neutralized 35% of heterologous clade B and 17% of global HIV isolates [9]. The HVTN 133 trial demonstrated that antibody mutations that take years to develop in people living with HIV (PLWH) can be induced by vaccination in months. Work is ongoing to expand the breadth and potency of induced bnabs by the design of boosting immunogens to target MPER sequences of contemporary global HIV strains.

CD4 binding site (CD4bs) bnAbs are both potent and broad and thus represent key vaccine targets. There are two types of CD4bs bnAbs, which include those that mimic CD4 binding through a gene‐restricted CDRH2 motif and others that utilize CDRH3 to bind the CD4bs [4]. CD4bs immunogens are based on HIV envelope (Env) proteins identified during natural infection or engineered to bind to either type of CD4bs bnAb precursor B cell lineages. These immunogens successfully activate and expand CD4 mimicking CD4bs naïve B cell precursors and intermediate antibodies in humanized mouse strains [10], non‐human primates [11, 12] and humans [13]. CDRH3‐mediated neutralizing antibody lineages have also been induced in the HVTN 300 trial using a stabilized germline‐targeting Env trimer [14]. CD4bs immunogens are being tested in a number of clinical trials including IAVI 002, HVTN 301, HVTN 320, HVTN 321, ACTG 5422 and IAVI C101.

V3‐glycan bnAbs require long CDRH3 segments or nucleotide insertion mutations and thus are disfavoured by the immune system. However, using germline‐targeting immunogens that bind to particular V3‐glycan bnAb naïve B cell precursors, V3‐glycan bnAb precursors have been induced in non‐human primates (NHPs) [15] and in humanized mice [16]. V3‐glycan bnAb targeting Envs are being tested in HVTN 144, HVTN 307 and HVTN 321 clinical trials.

V2 apex bnAbs also have long CDRH3s, and like V3‐glycan bnAbs, their precursors are relatively rare in the naïve B cell repertoire. Nonetheless, V2 apex immunogens have been designed that bind to certain V2 B cell precursor cells and have activated and expanded V2 apex bnAb lineages in humanized mice [17]. Immunogens that target the V2 apex are scheduled to be tested in HVTN trial HVTN 322.

The HIV fusion domain is expressed on the prefusion HIV Env and is a target for bnAb induction. HIV‐1 fusion domain vaccines include HIV‐1 fusion peptides arrayed on carrier molecules immunogens and will be tested in the NIH VRC trial, VRC020. BnAbs to the fusion domain have been induced in mice [18] and in monkeys [19], and fusion domain‐targeted bnAbs protect monkeys from Simian‐Human Immunodeficiency Virus (SHIV) infection [20], and immunogens have been tested in HVTN 303 and are schedule for testing in VRC020.

These recent successes have provided the proof‐of‐concept that bnAb lineages can be induced in animals and humans. It is clear that naïve B cell/germline targeting immunogens, followed by boosting with sequential immunogens, will be required to produce a bnAb‐based HIV vaccine. Other principles are that it will be necessary to induce multiple types of bnAbs to avoid HIV escape, that boosting immunogens will need to keep bnAb lineages on track and not induce competing off‐track antibodies, and that immunogens will need to induce durable bnAb responses. In addition, a successful immunogen may need to induce CD4+ T cell help and likely induce protective CD8+ T cells to eliminate any virions or virus‐infected cells that escape bnAb neutralizing activity [1].

To date, no immunization regimen has induced the types of neutralization responses required to achieve the degree of breadth and potency needed for consistent protection by a vaccine. Artificial intelligence algorithms trained to rapidly select Env mutants that will boost bnAb lineages to heterologous breath and potency may accelerate immunogen design. Thus, the road to a successful HIV vaccine is essentially to learn how to engineer the immune system to stimulate and mature rare neutralizing antibodies that infrequently occur in PLWH. Once accomplished, a multivalent immunogen will need to be formulated for a practical vaccine. While a difficult task, the rewards will be enormous by protecting those at risk from HIV acquisition and ending the HIV epidemic. Work is ongoing to design vaccine Env immunogens with many bnAb triggering sites on the same immunogen to minimize the number of vaccine components. Moreover, once the full set of rules are deciphered regarding the induction of disfavoured B cell responses, the same strategies can be applied to make other difficult‐to‐make vaccines. Thus, we can expect technologies developed in the HIV field to continue to enrich other fields as the HIV vaccine work progresses to success.

## COMPETING INTERESTS

BFH, GS, BHH, KW and KOS have patents on vaccine constructs discussed in this paper.

## AUTHORS’ CONTRIBUTIONS

BFH wrote the first draft of the paper. BBH, GMS, KW, KOS and LRB edited the paper. KW produced Figure 1.

## FUNDING

All authors were supported by HHS, NIH and NIAID UM1 grant AI144371 for the Consortia for HIV/AIDS Vaccine Development.

## Data Availability

Data sharing is not applicable to this article as no datasets were generated or analysed during the current study.
